# COGNITION AND HISTORY OF MULTIPLE HEALTH CONDITIONS OVER 20 YEARS: EVIDENCE FROM THE HEALTH AND RETIREMENT STUDY

**DOI:** 10.1093/geroni/igz038.1159

**Published:** 2019-11-08

**Authors:** Rebecca Bendayan, Alex D Federman, Richard J Dobson

**Affiliations:** 1 Dept. Biostatistics and Health Informatics, Institute of Psychiatry, Psychology & Neuroscience (IoPPN), King’s College London, London, United Kingdom, United Kingdom; 2 Division of General Internal Medicine, Department of Medicine, Icahn School of Medicine at Mount Sinai, New York, United States; 3 Dept. Biostatistics and Health Informatics, Institute of Psychiatry, Psychology & Neuroscience (IoPPN), King’s College London, London, United Kingdom

## Abstract

Research to date on multimorbidity and cognitive impairment is mainly cross-sectional or with limited history information of the health conditions. The present study explores the association between cognitive performance and previous history of health conditions over 24 years in a sample of 4858 respondents of the Health Retirement Study. Data from health conditions between 1998 and 2014 included self-reports for hypertension, diabetes, arthritis, stroke, cancer, lung and heart diseases and psychiatric problems. Duration of the health condition was categorized as more than 10 years, between 4 and 10 years, less than 4 years and no condition. Cognition was assessed using a summary index of cognitive performance including measures of memory, working memory, speed processing, knowledge, and language. ANOVA and post hoc tests were performed to explore the association between cognition and the duration of each health condition independently. Multiple linear regression analyses were performed to explore the association between multiple health conditions and cognitive performance. Results showed significant independent associations between cognitive performance in 2014 and each health condition, except for cancer [F(1,4)=2.60; p =.51]. When all the health conditions were considered in the regression models, we found that cognitive performance is negatively associated with high blood pressure and stroke (independently of the duration of the condition), long-term diabetes and lung diseases (i.e., for more than 10 years) and recent cancer (i.e., in the last 4 years). Our findings highlight that considering duration of co-existent health conditions is key for identifying individuals at greater risk of cognitive impairment.

